# Prioritizing geriatrics in medical education improves care for all

**DOI:** 10.1080/10872981.2022.2105549

**Published:** 2022-07-27

**Authors:** Samuel Rentsch, Caroline A. Vitale, Kahli Zietlow

**Affiliations:** a Department of Anesthesiology, University of Michigan, Ann Arbor, MI, USA; b Department of Medicine, University of Michigan and Geriatric Research Education and Clinical Center (GRECC), Veterans Affairs Healthcare System, Ann Arbor, MI, USA

**Keywords:** Geriatrics, older adults, Graduate Medical Education, Healthcare Disparities

## Abstract

Within the United States, there is a deficit of Geriatricians providing care for older adults, and this deficit will only grow as the population continues to age, meaning all clinicians, particularly Internal Medicine (IM) and Family Medicine (FM) trained physicians, will provide the bulk of primary care for older adults. However, geriatric training requirements for clinicians fall short, and in the case of IM were reduced as of 2022). Serving as a call to action, this article provides insight on ways to enhance geriatric education for all graduate medical trainees, utilizing both conventional teaching and newer, non-traditional media, such as national online journal clubs, podcasts, and online teaching curricula, as well as expanding sites of training to include evidence-based models of care, such as the Program of All-Inclusive Care for the Elderly (PACE). Providing geriatric education improves care for older adults and our future selves, ensuring trainees are prepared to care and advocate for this diverse and often vulnerable population.

The COVID-19 pandemic has exposed critical weaknesses in institutional systems intended to protect and promote public health[1]. In addition to exposing racial disparities, the pandemic has exposed pervasive ageism and systematic barriers that prevent older adults from accessing equitable, quality care. Older adults make up 15% of the U.S. population, yet, as of June 2022, accounted for 43% of hospitalizations and 75% of the mortality from COVID-19 (CDC.gov). Despite this increased burden of morbidity and mortality, the care of older adults has arguably not been prioritized by U.S. healthcare systems. Atypical presentations of COVID-19 have been overlooked, long-term care facilities remain under-resourced, and arbitrary trial exclusion criteria have left older adults without access to novel treatments[2]. In extreme cases, experts have advocated chronological age as an exclusion criteria for life-saving care[3]. While not new, COVID-19 has unmasked deeply entrenched and ugly truths within medicine.

To affect meaningful change, physician leaders equipped and well-versed in geriatric principles are desperately needed. Within the U.S., there are approximately 7,000 licensed Geriatricians, yet an estimated need of over 33,000. This leaves a staggering deficit of 26,000 physicians, which will only grow as the population ages[4]. With underrepresentation of Geriatricians, healthcare systems will need to rely on all clinicians to care for older adults. While Family Medicine (FM) and Internal Medicine (IM) physicians provide the bulk of care for older adults, training requirements for geriatric education are insufficient to match the needs of a rapidly aging population. FM training programs require 100 hours (e.g., one month of a 3 year long training experience) or 125 patient encounters dedicated to the care of the older adults. Furthermore, their operational age cutoff for an older adult is age 60, underrecognizing important nuances of caring for our oldest older adults[5].

Even more concerning, the Accreditation Council for Graduate Medical Education (ACGME) has *reduced* the geriatric education requirements for IM residents. Effective July 1^st^, 2022, IM residents will only be required to have an undefined clinical exposure or experience in Geriatric Medicine, a regression from the prior requirement of at least four weeks of dedicated geriatric experiences[6]. Moreover, these limited requirements only account for clinical training. IM and FM lack didactic requirements focusing on practical knowledge and skills relevant to the care of older adults, such as falls, deprescribing, healthcare finances, and/or guardianship [5,6]. This problem is not unique to the USA. Studies from across the globe highlight a lack of structured geriatric training and shortages of geriatricians to address rapidly aging populations [7–9].

These gaps become evident in the care older adults receive. In addition to the disproportionately negative outcomes of older adults with COVID-19, age portends increased risk of poor outcomes across healthcare settings. Post-operatively, older adults have higher rates of complications, readmissions, mortality, longer lengths of stay, and are more frequently discharged to post-acute care facilities, compared to adults under age 65 [10–14]. Close to 40% of older adults experience polypharmacy, which increases risk for adverse drug events, drug-drug interactions, and hospitalization[15]. On the other hand, some older adults are not considered for appropriate medication regimens, due to misplaced concerns about overprescribing that are inappropriate in healthy and highly functional patients[16]. Deeply embedded ageist constructs, such as the misconception that disability and/or poorer health are inevitable, are associated with poorer physical and mental health outcomes[17]. Furthermore, there is a paucity of literature examining how ageism intersects with other forms of discrimination that pervade our society, such as racism, homophobia, and sexism[18].

Although unintentional, the lack of targeted geriatric education and training among residency programs further exacerbates institutions’ inability to provide adequate care of this often-vulnerable population. Delirium and dementia are under-recognized, and risk being written off as normal aging. Sensory impairments preventing adequate communication are overlooked. Social factors, such as isolation, food insecurity, and lack of reliable internet access, often go unrecognized or worse, are disregarded. The under-recognition of geriatric syndromes and social determinants of health among older adults is associated with poorer quality of life, increased functional and cognitive impairment, medication non-adherence, poorer survival, increased healthcare utilization, and substantial costs to healthcare systems[19].

The ACGME Mission, Vision and Value statement is to ‘improve health care and population health by assessing and enhancing the quality of resident and fellow physicians’ education …’ [20] Programs accredited through ACGME train physicians who not only provide high quality care, but who also combat public health disparities and become future leaders in medicine. Yet we underrecognize our inadequacies towards caring for older adults. To turn the tide and enhance geriatric skillsets among future physicians, we strongly believe the ACGME can and should prioritize substantive changes to IM and FM residency training programs to enhance clinical and educational experiences in Geriatric Medicine.

Requirements for medical trainees seem to grow each year, and each specialty advocates for increased exposure and experiences. While we acknowledge adding additional curricular experiences is burdensome, we believe enhanced geriatric education is essential and can be feasibly done without undue strain on existing resources. IM/FM programs can intentionally incorporate geriatric curricula into existing frameworks, using innovative platforms for disseminating clinical principles and education. One such model is Project ECHO-Geriatrics, which, when utilized for geriatric conditions, has been shown to significantly increase resident competencies in geriatric-specified knowledge, and serves to disseminate geriatric education to remote clinical sites[21]. High quality online content through sites like Aquifer Geriatrics (aquifer.org/courses/aquifer-geriatrics), a national curriculum endorsed and supported by the American Geriatrics society, can provide supplemental education materials where geriatric expertise is not readily available[22]. There also exists a rapidly growing number of non-traditional educational media, such as podcasts like GeriPal (www.geripal.org) and Twitter Journal Clubs (https://twitter.com/geriatricsjc), which serve to enhance and disseminate geriatric educational content.

Additionally, we advocate for incorporating innovative models of care as required clinical rotation sites where available. Sites of evidence-based geriatric models of care, such as Program of All-inclusive Care for the Elderly (PACE) or Acute Care of Elderly (ACE) units, as well as post-acute and long-term care settings, provide unique opportunities for trainees to participate in the care of older adults under the tutelage of clinicians well-versed in Geriatric Medicine. Healthcare systems can also enact meaningful change by adopting policies and practices that support the care of older adults. Age-Friendly Health Systems utilize the ‘4 M’s’ of geriatrics: Medication, Mobility, Mentation, and (What) Matters Most, to incorporate each of these core principals into the older care adults receive[23]. Such initiatives inherently provide education to all providers and staff regarding these geriatric concepts, while leading to improved patient satisfaction and healthcare outcomes. The Institute for Healthcare Improvement provides a formal Age-Friendly designation for healthcare systems who formally incorporate the 4 Ms framework into the care provided[24].

The demographics are shifting and the need for access and exposure to geriatric role-models and mentors is critical. We cannot simply create vague requirements for ‘geriatric education’ and then allow important Geriatric clinical experiences and concepts to fade away. Geriatric experiences and representation within program leadership helps not only to ensure the future of the field, but to ensure we provide essential care to an aging population. The COVID-19 pandemic has illustrated the need for a collective and nonpartisan commitment for public health; we argue that this includes a commitment to medical training that proportionally accounts for the populations which we serve. This commitment calls for strategic, system-wide changes in how we deliver healthcare for older adults, starting with improved education and training to provide appropriate care targeted to the unique aspects of older adults and equipping our trainees with the skills necessary to competently care for older adults. Improving the care of older adults and building better healthcare will help everyone – including our current and future selves.
